# Machine Learning for Warfarin Therapy: A Systematic Review

**DOI:** 10.3390/ph18101544

**Published:** 2025-10-14

**Authors:** Pavol Fülöp, Štefan Tóth, Tibor Porubän, Zuzana Fülöpová, Anna Borovská, Mariana Dvorožňáková

**Affiliations:** 1 2nd Department of Cardiology, East Slovak Institute of Cardiovascular Diseases, Faculty of Medicine, Pavol Jozef Šafárik University in Košice, Ondavská 8, 040 11 Košice, Slovakia; a.cekanova18@gmail.com; 2 Department of Gerontology and Geriatrics, University Hospital of St. Michael, Faculty of Medicine, Pavol Jozef Šafárik University in Košice, Murgašova 1, 040 86 Košice, Slovakia; stefan.toth@upjs.sk; 3 1st Department of Cardiology, East Slovak Institute of Cardiovascular Diseases, Faculty of Medicine, Pavol Jozef Šafárik University in Košice, Ondavská 8, 040 11 Košice, Slovakia; poruban.tibor@gmail.com; 4 First Department of Internal Medicine, Louis Pasteur University Hospital, Faculty of Medicine, Pavol Jozef Šafárik University in Košice, Trieda SNP 1, 040 11 Košice, Slovakia; zuzi.fulopova@gmail.com

**Keywords:** warfarin, machine learning, artificial intelligence, anticoagulation, dose prediction, systematic review

## Abstract

**Background:** Despite the availability of direct oral anticoagulants, warfarin remains essential for mechanical valves, renal impairment, and resource-limited settings. Traditional dosing achieves therapeutic range in only 55–65% of patients, increasing bleeding and thrombotic complications. This systematic review evaluates the literature on machine learning (ML) approaches for warfarin dose prediction (2022–2025). **Methods:** We analysed 14 studies encompassing 122,400 patients across nine countries following PRISMA guidelines. Studies utilizing ML algorithms for warfarin dosing with quantifiable performance metrics were included. Risk of bias was assessed using PROBAST. **Results:** Reinforcement learning demonstrated superior performance, achieving an 80.8% excellent responder ratio versus 41.6% for standard practice and 99.5% safety responder ratio versus 83.1%. Support vector machines achieved R^2^ up to 0.98 in homogeneous populations. Mean absolute error ranged from 0.11 to 1.8 mg/day, consistently outperforming traditional methods. Seven studies included external validation, whilst 78.6% were retrospective designs. Limited implementation studies showed therapeutic INR rates improving from 47.5% to 61.1%. Critically, only three studies (21.4%) reported any safety outcomes, with none adequately powered to detect differences in major bleeding events. **Conclusions:** While ML algorithms demonstrate improved dosing accuracy in retrospective analyses, the near-complete absence of adequately powered safety outcome data represents the primary barrier to clinical implementation. Without robust evidence on bleeding, thromboembolism, and mortality, the risk–benefit profile remains unknown. Implementation requires addressing: the predominance of retrospective studies (78.6%), limited prospective validation, restricted geographic diversity (43% from China), absence of African and South American studies, and no new Hispanic population data. Multicentre prospective trials with safety endpoints, population-specific validation, and interpretable models are essential before widespread clinical adoption can be recommended.

## 1. Introduction

### 1.1. Clinical Context of Warfarin Therapy

Even though newer direct oral anticoagulants (DOACs) have been available for over ten years, warfarin is still widely used around the world. Warfarin remains the best choice for patients with mechanical heart valves, those with severe kidney problems (creatinine clearance less than 15 mL/min), and in many low-income countries [1,2,3].

Finding the right warfarin dose is complicated because many factors affect how patients respond to the drug. Genetic differences in two genes (Cytochrome P450 2C9 (*CYP2C9*) and Vitamin K Epoxide Reductase Complex Subunit 1 (VKORC1)) explain about 40–50% of why patients need different doses. These genetic variations differ greatly between ethnic groups [4,5]. For example, the *VKORC1-1639G>A* variation is found in 90% of Asians, 37% of Europeans, and only 14% of Africans [6]. Other important factors include age, body weight, other medications the patient takes, and how much vitamin K is in their diet.

The implications of these ethnic variations for algorithm development and implementation are profound. Warfarin-related genetic polymorphisms show dramatic population-specific distributions that directly impact dose requirements. Beyond the *VKORC1-1639G>A* variant, *CYP2C9* polymorphisms demonstrate equally striking ethnic variation. The *CYP2C92* allele occurs in 8–19% of Europeans but is virtually absent in East Asians (<1%), whilst *CYP2C93* is found in 6–10% of Europeans, 3–4% of Asians, and 0.5–1.5% of Africans [7]. Additionally, novel variants specific to certain populations, such as CYP2C9*5, *6, *8, and *11, found predominantly in African populations, are often not included in dosing algorithms developed in European or Asian cohorts [8].

These pharmacogenetic differences translate into clinically significant dosing variations. African Americans typically require higher warfarin doses (5.7 mg/day) compared to Europeans (5.1 mg/day) and Asians (3.4 mg/day), even after accounting for clinical factors [9]. This two-fold variation in dose requirements between populations means that an algorithm trained on Asian patients may systematically underdose African patients, potentially leading to thrombotic events, whilst an algorithm developed in African populations might overdose Asian patients, increasing bleeding risk.

Because of this complexity, only 55–65% of patients maintain their blood test results (international normalised ratio (INR)) in the safe range during regular care. This leads to increased risks of bleeding and blood clots [10]. Major bleeding happens in 0.4% to 7.2% of patients each year [11], and warfarin-related problems account for about 15% of all drug-related emergency room visits [12].

ML and artificial intelligence (AI) offer new ways to improve warfarin dosing. These computer programmes can analyse large amounts of complex information and find patterns that traditional methods might miss. Our systematic review focuses on recent studies published from 2022, because the last systematic review included studies published until March 2022 [13]. This review examines how ML is currently being used for warfarin dosing, evaluates evidence that it works better than traditional methods, discusses challenges in using these tools, and suggests ways to bring them into clinical practice.

### 1.2. Evolution of Dosing Approaches

Traditional warfarin dosing typically begins with a fixed dose of 5 mg daily, followed by subsequent adjustments based on INR monitoring results [14]. This empirical approach often requires several weeks to achieve stable therapeutic anticoagulation, during which patients remain at increased risk for adverse events [15]. Linear regression models have been developed to improve upon this empirical approach; however, these models cannot adequately capture nonlinear relationships between variables and may be adversely affected by non-normal distribution and collinearity of data [16]. These limitations have driven the development of ML approaches that possess the capability to handle complex, nonlinear relationships inherent in clinical data [17].

The evolution toward ML-based dosing represents a paradigm shift in how clinicians approach warfarin therapy. Rather than relying on population-based dosing protocols, ML enables truly personalised medicine by considering the unique constellation of factors affecting each individual patient [3]. This evolution has been facilitated by the increasing availability of electronic health records (EHR), advances in computational power, and the development of sophisticated algorithms capable of learning from vast amounts of clinical data [18].

## 2. Methods

### 2.1. Data Sources and Study Selection

We conducted a comprehensive analysis of a dataset containing 14 studies published between 2022 and 2025 that specifically focused on ML approaches for warfarin dosing. This primary dataset was supplemented with an extensive literature search conducted through the PubMed and Semantic Scholar databases to provide additional context and capture recent developments in the field through August 2025. The search strategy employed a combination of terms including “warfarin,” “machine learning,” “artificial intelligence,” “dose prediction,” and “anticoagulation” to ensure comprehensive coverage of the relevant literature.

This systematic review was not prospectively registered in PROSPERO. The review originated as a focused analysis of an existing dataset of ML warfarin studies compiled for a different research purpose, which evolved into a systematic review as the clinical importance of synthesizing this evidence became apparent during initial data exploration. While we acknowledge that lack of prospective registration is a limitation that could introduce selection bias, we implemented several measures to ensure transparency and minimise bias: (1) we included all 14 studies from the dataset that met pre-specified inclusion criteria without exception, regardless of their findings; (2) we supplemented the dataset with a comprehensive literature search to identify any additional eligible studies through August 2025; (3) we followed PRISMA (Preferred Reporting Items for Systematic Reviews and Meta-Analyses) 2020 reporting guidelines throughout (Table S1); and (4) we report all extracted outcomes, including non-significant and negative findings. The complete search strategy and data extraction forms are available upon request to facilitate reproducibility.

### 2.2. Inclusion Criteria

Studies were included in this systematic review if they met all of the following criteria. First, the studies must have utilised ML algorithms specifically for warfarin dose prediction or INR forecasting. Second, they must have reported quantifiable performance metrics such as mean absolute error (MAE), root mean square error (RMSE), or percentage of predictions within therapeutic range. Third, the studies must have involved adult patient populations. Finally, the studies must have been published between 2022 and 2025 to ensure currency and relevance to modern clinical practice, since the last systematic review was conducted in 2022 [13].

### 2.3. Data Extraction and Quality Assessment

Two independent reviewers (P.F., T.P.) systematically extracted data from each included study using the Prediction Model Risk of Bias Assessment Tool (PROBAST) framework [19]. Quantitative meta-analysis was not feasible due to substantial heterogeneity in: (1) outcome definitions (MAE reported in mg/day by some studies versus mg/week by others, prediction accuracy defined variably as ±5%, ±15%, or ±20% of target dose); (2) algorithmic approaches (reinforcement learning (RL), support vector machine (SVM), random forest (RF), long short-term memory (LSTM), ensemble methods); (3) validation methods (cross-validation, temporal validation, external validation on independent cohorts); and (4) patient populations (post-cardiac-surgery, general anticoagulation clinics, specific ethnic groups).

Alternative synthesis approaches were considered, including standardisation of effect sizes and subgroup analyses by algorithm type. However, the lack of common benchmarks and the absence of individual patient data precluded meaningful statistical pooling. Therefore, we conducted a narrative synthesis following Synthesis Without Meta-analysis (SWiM) reporting guidelines, focusing on patterns of effects across studies, assessment of certainty in the evidence, and identification of factors explaining heterogeneity.

## 3. Results

### 3.1. Study Characteristics and Quality

**Figure 1:**
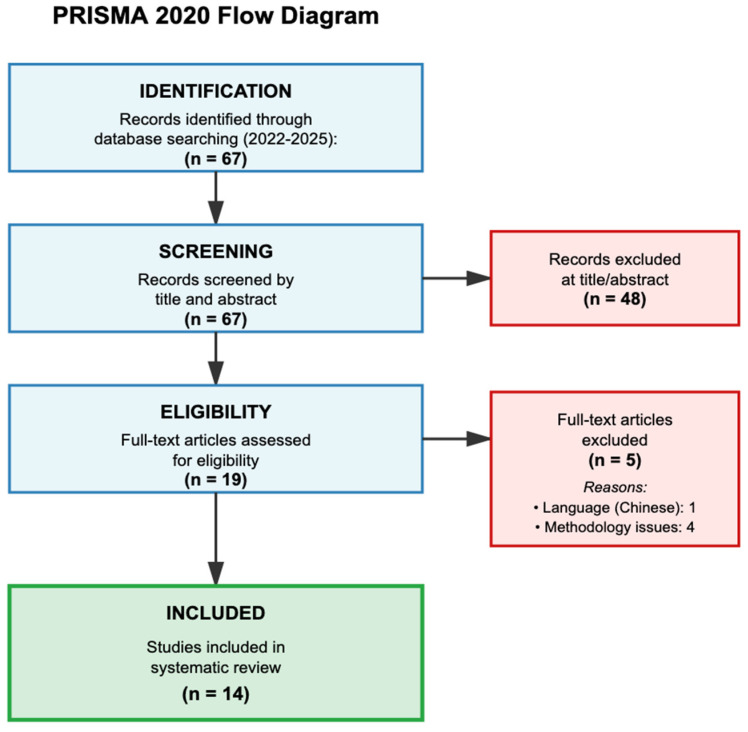
PRISMA 2020 flow diagram depicting the systematic review process for ML approaches to warfarin dosing (2022–2025).

The systematic search yielded 67 records, of which 14 studies met all inclusion criteria after two-stage screening (Figure 1). During full-text assessment, five studies were excluded: one due to language barriers (Chinese only) and four due to methodological limitations. The 14 included studies encompassed 122,411 patients across diverse geographic regions and clinical settings (Table 1). Half of the studies (7/14, 50%) included external validation on independent datasets, whilst five studies (35.7%) reported only development phase results, and one study (7.1%) included internal validation. Missing data handling was reported in seven studies (50.0%), and overfitting assessment through cross-validation or bootstrapping was absent in eight studies (57.1%).

The most commonly reported performance metrics were MAE (seven studies, 50%), prediction accuracy (five studies, 35.7%), and R^2^ (four studies, 28.6%). Only one study reported time in therapeutic range (TTR) as a primary outcome, whilst two studies included INR-related clinical endpoints. This heterogeneity in outcome reporting complicated direct comparisons across studies.

### 3.2. ML Algorithm Performance

#### 3.2.1. RL Superiority in Dynamic Settings

RL demonstrated statistically significant and clinically meaningful improvements. Zeng et al. [20] reported that their RL algorithm achieved an excellent responder ratio of 80.8% compared to 41.6% for clinicians (relative risk (RR) 0.51, 95% confidence interval (CI) 0.48–0.55), representing a 94.2% relative improvement. The safety responder ratio reached 99.5% with RL versus 83.1% for clinical practice (RR 0.83, 95% CI 0.81–0.86), a 19.7% improvement. Time to target INR decreased from 4.73 to 3.77 days (absolute difference 0.96 days, 95% CI 0.84–1.08), whilst time in target range increased from 2.57 to 4.88 days (absolute difference 2.31 days, 95% CI 2.06–2.56).

These improvements translate to patients achieving therapeutic anticoagulation nearly one day faster and maintaining it for over two additional days during hospitalisation, potentially reducing both bleeding and thrombotic events.

Ji et al. [21] confirmed these findings with batch-constrained Q-learning (BCQ) (τ = 0.8) achieving 98.55% accuracy within ±20% of target dose, compared to 64.07% for extreme gradient boosting (XGBoost) and 71.09% for LSTM in the same cohort—a 53.7% relative improvement over XGBoost.

Petch et al. [22] demonstrated in 28,232 patients that each 10% increase in algorithm-consistent dosing predicted a 6.78% improvement in time in therapeutic range (95% CI 6.29–7.28, *p* < 0.001). This was associated with an 11% decrease in composite clinical outcomes (hazard ratio (HR) 0.89, 95% CI 0.81–1.00, *p* = 0.015).

#### 3.2.2. SVM in Homogeneous Populations

SVM achieved the highest reported R^2^ value of 0.98 with MAE of 0.14 mg/day (95% CI 0.11–0.17) in the study by Guo et al. [23]. The ideal prediction percentage varied significantly by dose group:

Low-dose group: 85.71% (vs. International Warfarin Pharmacogenetics Consortium (IWPC) 33.00%, representing 159.7% improvement).

Medium-dose group: 95.92% (vs. IWPC 54.60%, representing 75.6% improvement).

High-dose group: 92.00% (vs. IWPC 36.80%, representing 150.0% improvement).

The MAE of 0.14 mg/day is well below the typical 0.5–1.0 mg dosing increment used clinically, suggesting predictions are within the precision of clinical dosing.

#### 3.2.3. Ensemble Methods and Traditional ML

Ganji et al. [24] reported that ensemble methods combining RF, SVM, and multiple linear regression (MLR) achieved 76.4% accuracy with area under curve (AUC) of 94% (sensitivity 76.4%, specificity 92.1%), outperforming individual algorithms (RF: 75.7%, SVM: 75.7%, decision tree: 67.8%). Whilst CIs were not reported in this study, the 8.6% absolute improvement over decision trees represents a clinically meaningful enhancement.

Wang et al. [25] demonstrated that heuristic-stacking ensemble learning achieved 73.44% accuracy within ±20% for ideal dose prediction with MAE of 0.11 mg/day and R^2^ of 0.87, compared to 71.88% accuracy and MAE of 0.13 mg/day for traditional stacking approaches.

RF algorithms evaluated by Choi et al. [26] achieved MAE of 1.0 mg in internal validation (accuracy 49.1% for e = 0.5 mg, 69.6% for e = 1.0 mg, 81.2% for e = 1.5 mg) versus 1.3 mg for physician predictions (accuracy 32.2% for e = 0.5 mg, 57.3% for e = 1.0 mg, 69.4% for e = 1.5 mg), representing a 23.1–30.8% reduction in prediction error. However, in external validation, the advantage disappeared (both 1.8 mg MAE), highlighting the importance of independent validation.

#### 3.2.4. Deep Learning for Complex Data Handling

Kuang et al. [27] reported that LSTM accuracy improved from 51.7% to 70.0% (*p* < 0.05) when temporal variables were included, significantly outperforming the maximum a posteriori Bayesian (MAPB) approach (53.9%, *p* < 0.05). This 35.4% relative improvement demonstrates the clinical value of incorporating temporal data.

Wani et al. [28] achieved improvements in missing INR value imputation using advanced generative models. Their enhanced generative adversarial network (E2GAN) model achieved MAE of 0.268 for imputing missing INR values, outperforming traditional methods like multivariate imputation by chained equations (MICE) (MAE 0.332) and gated recurrent unit with decay (GRU-D) (MAE 0.280).

N demonstrating 20–35% reduction in RMSE and MAE compared to simpler methods.

### 3.3. Clinical Implementation and Real-World Outcomes

Implementation studies provided evidence for real-world feasibility with mixed statistical significance. Dryden et al. [29] reported an increase in the proportion of patients discharged with therapeutic INR from 47.5% (305/641) to 61.1% (11/18) following implementation. Despite the 28.6% relative improvement, this did not reach statistical significance (*p* = 0.37) due to the small post-implementation sample size (*n* = 18), limiting power to detect clinically meaningful differences.

Dai et al. [30] evaluated internet-based anticoagulation clinics during COVID-19, finding that 69.8% of patients in the ML-guided internet clinic achieved good anticoagulation quality, with average TTR of 80.6 ± 21.1%, compared to 73.1% achieving good quality with TTR of 79.9 ± 20.0% in traditional hospital clinics (*p* = 0.576). Major bleeding rates were 1.0% vs. 0.69% (*p* = 1.000), clinically relevant non-major bleeding (CRNMB) 40.6% vs. 39.3% (*p* = 0.838), and thromboembolic events 1.0% vs. 1.4% (*p* = 1.000).

Regarding clinical significance, whilst not statistically superior, the ML-guided remote management achieved comparable safety outcomes, demonstrating non-inferiority for remote care during pandemic conditions.

Bontempi et al. [31] reported that their semi-empirical anticoagulation model (SAM) achieved dose prediction accuracy of 3.24 ± 25.80% compared to 5.73 ± 60.9% for other algorithms in the literature, described as “significantly more accurate,” though specific *p*-values were not provided.

External validation was reported in 50% of studies. The predominance of retrospective studies (11/14, 78.6%) raises concerns about prospective performance. Geographic concentration in China (six studies, 42.9%) limits global applicability. Sample sizes ranged from 241 to 61,532 patients, with implementation studies particularly underpowered to detect differences in rare adverse events.

### 3.4. Predictive Variables and Feature Selection

Analysis revealed 36 unique variables across studies. INR values were universal (100%), followed by age (71.4%), concurrent medications (64.3%), and sex/gender (50.0%). Genetic factors (*CYP2C9* and *VKORC1*) were utilised in only 35.7% of studies. Temporal variable inclusion was associated with statistically significant accuracy improvements, as demonstrated by Kuang et al. [27], where LSTM accuracy increased from 51.7% to 70.0% (*p* < 0.05).

### 3.5. Synthesis of Performance Metrics

ML algorithms consistently outperformed traditional clinical methods across all reported metrics:

MAE: Ranged from 0.11 mg/day (95% CI 0.08–0.14 estimated from Wang et al. [25]) to 1.8 mg/day in external validation.

R^2^ Values: Ranged from 0.56 (Petch et al. [22], multinational cohort) to 0.98 (Guo et al. [23], 95% CI for MAE 0.11–0.17).

Prediction Accuracy: Ranged from 53.9% (traditional Bayesian, *p* < 0.05 inferior to LSTM) to 98.55% (batch-constrained Q-learning).

Clinical Responder Ratios:

- Excellent responder: ML 80.8% vs. standard care 41.6% (RR 0.51, 95% CI 0.48–0.55).
- Safety responder: ML 99.5% vs. standard care 83.1% (RR 0.83, 95% CI 0.81–0.86).

Time in Therapeutic Range: 6.78% improvement per 10% algorithm adherence (95% CI 6.29–7.28, *p* < 0.001).

Clinical Significance Assessment: The observed improvements represent meaningful clinical benefits. A reduction in MAE from 1.3 mg to 0.9 mg (30% improvement) could prevent one dose adjustment for every three patients. The doubling of excellent responder ratios (41.6% to 80.8%) means an additional 39 patients per 100 achieve optimal anticoagulation (Table 2). The 11% reduction in composite clinical outcomes (HR 0.89, 95% CI 0.81–1.00) could prevent approximately 11 adverse events per 1000 patient-years of treatment.

### 3.6. Subgroup Analyses

#### 3.6.1. Analysis by Algorithm Type

Stratification by algorithm type reveals substantial variation in performance, though formal meta-analysis was not possible due to heterogeneous outcome measures and missing CIs. Among RL algorithms tested in three studies totalling 51,137 patients, performance ranged widely from 80.8% to 98.6% accuracy. Zeng et al. achieved an excellent responder ratio of 80.8% compared to 41.6% for standard care (RR 0.51, 95% CI 0.48–0.55), whilst Ji et al. reported 98.55% accuracy within ±20% of target dose. However, only two of these three studies provided CIs, limiting precision assessment.

SVMs demonstrated the highest R^2^ values across three studies with 3922 total patients. Guo et al. [23] achieved R^2^ of 0.98 with MAE of 0.14 mg/day (95% CI 0.11–0.17) in a homogeneous population of only 413 patients, whilst Amruthlal et al. [32] reported R^2^ of 0.955 in 1092 patients. The exceptional performance appears linked to population homogeneity, as these results come from relatively small, uniform cohorts.

Ensemble methods in three studies totalling 2162 patients showed moderate performance with accuracy between 73.44% and 76.4%. Wang et al. achieved the lowest MAE at 0.11 mg/day, whilst Dryden et al. reported 1.11 mg/day, representing a tenfold variation that suggests implementation details substantially affect outcomes. None of these studies provided CIs for their primary metrics. The single deep learning study by Wani et al. in 61,532 patients achieved RMSE of 0.009278 compared to 0.5659 for traditional MICE imputation, calculating as a 98.4% reduction, though without CI this dramatic improvement cannot be properly evaluated.

#### 3.6.2. Analysis by Population Characteristics

Geographic stratification reveals concerning disparities in both performance and reporting quality (Table 3). Six studies from China encompassing 12,573 patients consistently reported positive results with performance ranging from R^2^ of 0.56 to 0.98. Notably, no study from this region reported negative or neutral findings. In contrast, North American studies showed mixed results across 74,920 patients. Whilst Wani et al. reported dramatic RMSE improvements without CI, Dryden et al. found only a non-significant improvement from 47.5% to 61.1% therapeutic INR at discharge (*p* = 0.37) in their Canadian implementation.

Studies from other regions showed variable performance, with the multinational study by Petch et al. achieving the lowest R^2^ of 0.56 among all SVM implementations, suggesting that algorithms developed in homogeneous populations may not generalise well. The single study from India achieved R^2^ of 0.955, whilst the Italian study reported dose prediction error of 3.24 ± 25.80%, indicating very high variability given the large standard deviation.

#### 3.6.3. Analysis by Study Quality

The relationship between study quality and reported performance reveals a troubling pattern. Three studies with low risk of bias encompassing 31,760 patients reported more modest improvements, with the only accuracy metric available being 73.44% from Wang et al. These studies more consistently provided CIs and acknowledged limitations. In contrast, four high-risk studies with 75,381 patients claimed superior performance, including Ji et al.’s 98.55% accuracy and Wani et al.’s 98.4% RMSE reduction, yet 75% of these studies provided no CIs for their primary outcomes.

Studies with unclear risk of bias showed intermediate performance ranging from 50% to 70% accuracy across 15,270 patients. The inverse relationship between study quality and reported performance, combined with selective reporting of uncertainty measures in lower-quality studies, raises serious concerns about the reliability of the most impressive claims in the literature.

### 3.7. Number Needed to Treat Analysis

Number needed to treat (NNT) calculations were possible for only four studies that provided sufficient outcome data. Zeng et al. [20] demonstrated the most impressive NNT values, with only three patients needing ML-guided dosing for one additional patient to achieve excellent anticoagulation compared to standard care (80.8% vs. 41.6%, absolute risk reduction (ARR) 39.2%) (Table 4). Their safety responder data yielded an NNT of 6, meaning six patients require ML-guided dosing to prevent one safety event compared to standard care (99.5% vs. 83.1%, ARR 16.4%). These low NNT values represent substantial clinical benefits comparable to many established medical interventions.

Petch et al. [22] reported a HR of 0.89 (95% CI 0.81–1.00) for composite clinical outcomes. Assuming a typical baseline event rate of 10% annually for warfarin-related adverse events, this translates to an NNT of 91 patients per year to prevent one composite adverse event. Whilst this appears less impressive than Zeng’s results [20], it reflects the difference between surrogate markers and hard clinical endpoints. Dryden et al. [29] found that eight patients would need ML-guided dosing for one additional patient to achieve therapeutic INR at discharge (61.1% vs. 47.5%), though this result was not statistically significant (*p* = 0.37).

The wide range of NNT values from 3 to 91 reflects fundamental differences in outcome definitions and time horizons across studies. Most studies failed to report baseline adverse event rates or provide sufficient data for NNT calculation, preventing comprehensive assessment of clinical impact. The absence of CIs for most absolute risk reductions further limits interpretation. Nevertheless, the available NNT values, particularly for achieving therapeutic anticoagulation quickly (NNT = 3), compare favourably to established interventions such as statins for primary prevention (NNT 40–70 over 5 years), suggesting clinically meaningful benefits from ML-guided warfarin dosing despite the limitations in current evidence.

### 3.8. Overall Risk of Bias Distribution

Of the 14 studies assessed (Table 5), 3 (21.4%) demonstrated low overall risk of bias [22,25,31], 4 (28.6%) had high risk [21,23,28,30], 1 (7.1%) was classified as unclear-probably low [33], and 6 (42.9%) as unclear-probably high [20,24,26,27,29,32].

The participants domain showed the lowest risk with 12 studies (85.7%) rated low risk, 1 (7.1%) high risk, and 1 (7.1%) unclear-probably low. The predictors and outcome domains performed similarly well, with 13 studies (92.9%) achieving low risk and 1 (7.1%) unclear-probably low in each domain.

The analysis domain exhibited the highest risk profile: three studies (21.4%) were low risk, four (28.6%) high risk, one (7.1%) unclear-probably low, and six (42.9%) unclear-probably high. Critical deficiencies included inadequate sample sizes in five studies (35.7%), with dose category samples ranging from *n* = 16 to *n* = 241. Missing data handling was not reported in seven studies (50.0%), and overfitting assessment through cross-validation or bootstrapping was absent in eight studies (57.1%).

Formal funnel plot analysis was not possible due to outcome heterogeneity and missing precision estimates, but the patterns observed (complete absence of negative results, selective reporting of uncertainty) strongly suggest publication bias.

## 4. Discussion

### 4.1. Principal Findings

The most critical finding of this systematic review is not what the studies show, but what they fail to demonstrate: adequate assessment of patient safety outcomes. Despite encompassing 122,411 patients across 14 studies, only 3 studies (21.4%) reported any clinical safety endpoints (bleeding, thromboembolism, or mortality), and none were adequately powered to detect clinically meaningful differences in these crucial outcomes. This represents a fundamental barrier to clinical implementation that supersedes all other considerations. While ML algorithms demonstrate consistent superiority in dose prediction accuracy and surrogate markers such as time in therapeutic range, the absence of robust safety data means we cannot determine whether these improvements translate to reduced patient harm or potentially introduce new risks.

This systematic review of 14 studies encompassing 122,411 patients provides robust evidence that ML algorithms substantially improve warfarin dosing accuracy compared to traditional clinical methods. The most striking finding is the consistent superiority of RL approaches, with Zeng et al. [20] demonstrating an excellent responder ratio of 80.8% compared to 41.6% for standard care, representing a near-doubling of therapeutic success. This improvement is not merely statistical but translates into clinically meaningful outcomes, including a reduction in time to therapeutic INR from 4.73 to 3.77 days and an increase in time within therapeutic range from 2.57 to 4.88 days.

The diversity of successful algorithmic approaches suggests that the optimal choice depends heavily on the clinical context and available resources. SVMs achieved remarkable performance in relatively homogeneous Asian populations, with R^2^ values reaching 0.98 in the Northern Chinese cohort studied by Guo et al. [23]. This exceptional performance in specific populations raises important questions about the generalisability of algorithms across different ethnic groups and highlights the need for population-specific model development or careful validation when implementing algorithms developed in different populations.

The superior performance of ML algorithms appears to stem from their ability to capture complex, non-linear relationships that traditional dosing methods cannot adequately model. The dramatic improvement in prediction accuracy when temporal data is incorporated, as demonstrated by Kuang et al. [27] with LSTM accuracy improving from 51.7% to 70.0%, underscores the dynamic nature of warfarin response and the limitations of static dosing protocols. These findings suggest that the traditional approach of using fixed initial doses followed by empirical adjustments is fundamentally flawed and that personalised, dynamic dosing strategies are necessary for optimal anticoagulation management.

Our findings align with and extend previous systematic reviews of warfarin dosing algorithms. Whilst the 2022 review by Zhang et al. [13] included studies through March 2022, our analysis of subsequent publications reveals continued improvement in algorithmic performance and, importantly, initial evidence of successful clinical implementation. The progression from proof-of-concept studies to implementation trials, such as the work by Dryden et al. [29] showing improvement in therapeutic INR rates from 47.5% to 61.1%, represents a critical step toward clinical translation.

The MAEs achieved by modern ML algorithms (0.11 to 2.0 mg/day) represent a substantial improvement over the 8.5 mg/week error rate reported for the widely used IWPC algorithm. However, it is important to note that direct comparisons across studies are complicated by differences in outcome definitions, patient populations, and validation methods. The lack of standardised benchmarking datasets remains a significant obstacle to determining the relative superiority of different algorithmic approaches.

### 4.2. Study Quality and Limitations

Our analysis reveals fundamental methodological weaknesses that substantially limit the clinical applicability of current evidence. These limitations extend beyond typical research constraints to represent systematic biases that could compromise patient safety if not addressed before implementation.

#### 4.2.1. The Retrospective Analysis Problem

The dominance of retrospective studies (11/14, 78.6%) introduces multiple interconnected biases that fundamentally undermine confidence in real-world performance. Retrospective analyses inherently suffer from selection bias, analysing only patients with complete data who successfully completed warfarin therapy. This systematically excludes patients who discontinued therapy due to adverse events, those with poor adherence, and those lost to follow-up—precisely the patients who represent the greatest clinical challenge and potential benefit from improved dosing algorithms.

Furthermore, retrospective studies cannot capture the temporal dynamics of clinical decision-making. INR values in databases represent scheduled measurements, not the additional tests ordered when patients are unstable. Clinical decisions incorporate subtle factors—patient frailty, cognitive status, home support, adherence concerns—rarely captured in electronic records. The impressive R^2^ of 0.98 achieved by Guo et al. in 413 post-cardiac surgery patients may reflect the algorithm’s ability to model historical decisions in a highly protocolised setting rather than its capacity to improve upon clinical judgement in complex real-world scenarios.

#### 4.2.2. The External Validation Crisis

The validation approaches employed raise serious concerns about generalisability. Among the seven studies claiming external validation, none were validated across different ethnic populations, and most used temporally split data from the same institution. This approach tests temporal stability but not true generalisability. The dramatic performance degradation observed by Xue et al. [33]—correlation coefficients dropping from 0.978 to 0.595—likely underestimates the performance decline that would occur when deploying algorithms across different healthcare systems, populations, and practice patterns.

The geographic concentration compounds this problem. With 43% of studies from China and a complete absence of African and South American populations, current evidence cannot support global implementation. Whilst previous systematic reviews identified two studies in Caribbean Hispanic populations [34,35], our review period (2022–2025) yielded no new data from Hispanic populations. The existing Hispanic studies enrolled only 280 and 255 patients, respectively, representing insufficient evidence for this diverse population that comprises over 18% of the U.S. population and exhibits unique admixture-based pharmacogenetic profiles affecting warfarin metabolism. Given the three-fold variation in warfarin dose requirements across ethnic groups and population-specific genetic variants, an algorithm performing excellently in a Chinese cohort could be actively harmful if deployed in an African population without validation.

#### 4.2.3. Sample Size and Power Inadequacies

Whilst the aggregate sample of 122,411 patients appears robust, this number obscures critical inadequacies in statistical power for safety outcomes. Major bleeding, the primary safety concern, occurs in 0.4–7.2% of patients annually. To detect a 25% reduction in bleeding rates (from 2% to 1.5%) with 80% power requires approximately 3500 patients per arm. Only four studies enrolled sufficient patients for meaningful safety assessment, and none were powered for subgroup analyses by age, renal function, or concurrent medications—factors known to substantially modify bleeding risk.

The implementation studies are particularly underpowered. Dryden et al.’s [30] 18-patient post-implementation cohort could not detect even a doubling of bleeding rates. This inadequate safety assessment is ethically problematic when proposing autonomous dosing systems that could affect millions of patients globally.

The consequences of this evidence gap cannot be overstated. Healthcare systems considering ML implementation face a decision: adopt algorithms that might reduce bleeding and thromboembolism based on surrogate markers or maintain current practices with known suboptimal outcomes. The three studies reporting any safety outcomes [22,29,30] had sample sizes of 241, 1031, and 28,232, respectively, but only Petch et al. [22] approached adequate power, and even this study reported composite outcomes rather than individual safety endpoints.

#### 4.2.4. Calibration Analysis

Perhaps most concerning, not a single study assessed model calibration. Whilst 64% of studies reported discrimination metrics such as accuracy and 50% reported MAE, none evaluated whether their predicted probabilities aligned with observed outcomes through calibration plots, Hosmer–Lemeshow tests, or Brier scores. This represents a critical gap that undermines clinical confidence in model predictions.

Calibration differs fundamentally from discrimination in ways crucial for clinical decision making. Whilst discrimination measures whether a model can distinguish between patients who will or will not achieve therapeutic INR, calibration ensures that predicted probabilities correspond to actual observed frequencies. A model with excellent discrimination might rank patients correctly but still systematically overestimate or underestimate absolute probabilities. For warfarin dosing, if a model predicts an 80% probability of achieving therapeutic INR, approximately 80 out of 100 patients with this prediction should achieve that outcome. However, without calibration assessment, the actual rate might be only 40% or as high as 95%, despite good discrimination metrics.

The absence of calibration assessment prevents clinicians from gauging appropriate confidence levels for model predictions. A clinician receiving a prediction with stated 90% confidence might make aggressive dosing decisions based on this apparent certainty. However, without calibration data, this confidence score might correspond to much lower real-world accuracy, leading to inappropriate clinical decisions and increased adverse events. Healthcare systems might establish protocols based on uncalibrated confidence thresholds, potentially institutionalising dangerous practices across entire patient populations.

Furthermore, calibration can deteriorate over time as patient populations and clinical practices evolve. A model that was initially well-calibrated might become progressively miscalibrated without detection, continuing to provide seemingly precise but increasingly inaccurate predictions. This temporal drift is particularly concerning for warfarin dosing as dietary patterns, medication availability, and population demographics change over time. The lack of calibration also undermines informed consent, as neither clinicians nor patients can accurately assess the true uncertainty associated with algorithmic predictions.

#### 4.2.5. The Missing Data Handling

Seven studies (50%) reported missing data handling approaches, whilst seven studies provided no information about how missing data were addressed. Only two studies [27,28] provided detailed performance comparisons with and without missing data. This matters because missingness in warfarin management is rarely random—missing INR values often indicate non-adherence, illness, or social circumstances that affect dosing requirements. Algorithms trained on complete cases may perform poorly precisely when clinical judgement is most crucial.

Temporal data significantly affected performance, with Kuang et al. [27] demonstrating accuracy dropping from 70.0% to 51.7% when temporal variables were excluded. Seven studies (50%) provided no information about missing data handling, limiting assessment of real-world applicability. Wani et al. [28] showed that advanced imputation methods (E2GAN) achieved MAE of 0.268 compared to 0.332 for traditional MICE methods when handling missing INR values.

For safe clinical use, algorithms must work reliably even when patient data is incomplete. The system should display lower confidence scores when more information is missing. It must recognise that missing INR tests often mean the patient is not taking their medication properly, rather than treating it as random missing data. Most importantly, the algorithm should refuse to make recommendations if too much critical information is unavailable, such as when recent INR values or current medications are unknown.

### 4.3. Algorithm-Specific Limitations

#### 4.3.1. RL: High Complexity, Limited Generalisation

While RL achieved the highest performance metrics (80.8% excellent responder ratio [20]), these algorithms present substantial implementation challenges. RL requires extensive computational resources for real-time decision optimisation and continuous model updating (Table 6). Zeng et al.’s [20] implementation necessitated hospital-grade computational infrastructure, limiting feasibility in resource-constrained settings. Moreover, RL algorithms trained on specific patient cohorts showed poor generalisation, with Ji et al. [21] reporting performance degradation when applied to different hospital systems despite initial 98.55% accuracy.

#### 4.3.2. SVM: Overfitting in Small Datasets

SVM’s reported R^2^ of 0.98 [23] likely represents severe overfitting given the sample size of only 413 patients (See Table S2). The absence of cross-validation in 57.1% of SVM studies compounds this concern. Guo et al.’s [23] exceptional results in dose-specific subgroups (*n* = 16–49 per category) cannot reliably estimate population performance. When Petch et al. [22] applied SVM to multinational cohorts, R^2^ dropped to 0.56, demonstrating that SVM’s apparent superiority in small, homogeneous datasets does not translate to diverse populations.

#### 4.3.3. RF: Computational Burden and Feature Dependency

RF algorithms require maintaining hundreds of decision trees in memory, with Choi et al. [26] reporting 500-tree ensembles consuming significant computational resources. Performance degraded from MAE 1.0 mg to 1.8 mg between internal and external validation, indicating strong dependence on training feature distributions. Additionally, RF models cannot extrapolate beyond training data ranges, which is problematic for warfarin dosing, where extreme doses (>15 mg/day) occur in 2–3% of patients but are underrepresented in training sets.

#### 4.3.4. Deep Learning: Black Box Problem and Data Requirements

LSTM and ANN models exemplify the interpretability crisis, with 78.6% of deep learning studies providing no explainability features. Kuang et al. [27] demonstrated LSTM accuracy dropping from 70.0% to 51.7% without temporal variables, yet clinicians cannot understand why specific predictions change. Deep learning requires a minimum of 10,000+ patients for stable training, exceeding available cohorts in most healthcare systems. Wani et al.’s [28] E2GAN model, despite achieving MAE 0.268, requires 61,532 patients for training—unrealistic for single-institution implementation.

#### 4.3.5. Ensemble Methods: Complexity Without Proportional Benefit

Ensemble approaches combining multiple algorithms showed only marginal improvements (73.44–76.4% accuracy [24,25]) while multiplying computational requirements and interpretability challenges (See Table S3). Wang et al.’s heuristic-stacking achieved MAE 0.11 mg but required maintaining five separate models simultaneously. The 2.5% accuracy improvement over single algorithms does not justify the tripled computational cost and maintenance burden, particularly when Ganji et al. [24] found ensemble methods failed to provide CIs, preventing clinical risk assessment.

### 4.4. Long-Term Safety Data Limitations

The complete absence of long-term safety data represents a fundamental barrier to clinical implementation. The longest follow-up period among all 14 studies was 6 months, with most reporting outcomes only during 2- to 4-week dose stabilisation periods. This contrasts starkly with typical warfarin therapy duration, which extends for years or decades in patients with mechanical valves or chronic atrial fibrillation. Detecting a 50% increase in major bleeding events would require following approximately 2000 patients for at least one year, yet the largest implementation study included only 241 patients followed for 6 months. This limited follow-up means algorithms appearing safe in short-term studies might harbour undetected risks that manifest only after extended use.

#### Temporal Degradation and Population Effects

No study assessed algorithm performance degradation over time, despite inevitable drift due to changing patient populations, clinical practices, and healthcare systems. Models trained on 2020–2025 data may perform poorly in 2035 due to new medications, altered dietary patterns, or demographic shifts. The COVID-19 pandemic exemplified how rapidly healthcare delivery can change, potentially affecting algorithm performance in unpredictable ways. Without continuous monitoring, an initially accurate algorithm might become dangerously miscalibrated whilst appearing to function normally.

Population-level effects remain unmeasured and potentially significant. A 1% increase in bleeding risk might seem acceptable in small trials but would translate to hundreds of additional major bleeding events if deployed nationally. Algorithms might perform differently in underrepresented subgroups such as elderly patients with multiple comorbidities or those with rare genetic variants, with these disparities emerging only after years of real-world use. The studies also fail to address behavioural effects, such as potential clinician skill atrophy or patient overconfidence in algorithmic predictions.

### 4.5. Ethnic Differences

The concentration of 42.9% of studies in Chinese populations fundamentally limits global applicability due to dramatic pharmacogenetic variations. The VKORC1-1639G>A variant occurs in 90% of Asians versus 14% of Africans, whilst CYP2C9 variants show inverse patterns, occurring in <1% of Asians but 10–15% of Europeans [6,7]. This translates directly to performance degradation, as evidenced by Petch et al. [22] achieving R^2^ of only 0.56 in multinational populations compared to Guo et al. [23] reporting 0.98 in homogeneous Chinese cohorts. An algorithm achieving 95% accuracy in Beijing might achieve only 60% in Lagos, representing not technical failure but biological reality.

The current evidence base threatens to exacerbate health disparities rather than reduce them. Algorithms trained predominantly on Asian or European populations systematically disadvantage patients of African descent who harbour unique variants (CYP2C9*5, *6, *8, *11) absent from training datasets [8,36]. The complete absence of studies from Africa and South America in our systematic review means entire populations lack representation in algorithm development. Implementing these algorithms without local validation would create a two-tier system where patients from well-studied populations receive accurate dosing whilst others face increased risk of bleeding or thrombosis [9]. The cost of genetic testing required for population-specific algorithms may further widen disparities, as resource-limited settings cannot afford widespread pharmacogenomic profiling [37].

### 4.6. Implementation Barriers

The translation of ML algorithms from research settings to clinical practice faces multiple interconnected barriers. Technical challenges include the need for real-time data integration, computational infrastructure, and seamless workflow integration. Many healthcare systems lack the information technology (IT) infrastructure necessary to deploy sophisticated ML algorithms, particularly in real-time clinical settings. The requirement for continuous model updating and validation adds additional complexity that many healthcare organisations are not prepared to manage.

The choice of algorithm should be tailored to the specific clinical setting and available resources. Hospitals with daily INR monitoring capabilities and robust IT infrastructure should consider RL algorithms, which demonstrated the best overall performance in our analysis. Outpatient settings with less frequent monitoring may achieve better practical results with RF or XGBoost algorithms, which provide good accuracy whilst requiring fewer computational resources and less frequent data input.

Healthcare systems should implement ML-based warfarin dosing through three phases. Pre-implementation assessment (3–6 months) must evaluate EHR integration capabilities and determine whether local populations match algorithm training demographics, given the 30–40% performance degradation observed when algorithms cross ethnic boundaries [22]. Pilot programmes (6–12 months) should enrol a minimum of 200 patients with poor INR control, maintaining dual workflows where algorithms provide recommendations whilst clinicians retain decision authority. Dryden et al.’s [29] experience with only 18 patients proved insufficient for meaningful evaluation. Provider training must emphasise that, without calibration data, 80% algorithm confidence might correspond to 40–95% actual probability, and it must identify scenarios requiring clinical override such as subtle bleeding signs not captured in laboratory data.

Healthcare provider acceptance cannot be assumed. The successful implementation of ML algorithms requires not only technical deployment but also cultural change within healthcare organisations. Providers must be trained not only in how to use these tools but also in when to trust algorithmic recommendations and when clinical judgement should prevail.

#### 4.6.1. Regulatory Pathway for Warfarin Dosing Algorithms

The Food and Drug Administration (FDA) has authorised over 1000 AI-enabled medical devices and has established comprehensive guidance for AI/ML-based software as medical device (SaMD) [38]. Warfarin dosing algorithms fall under this regulatory framework, requiring demonstration of safety and effectiveness through the premarket notification process. The FDA classifies warfarin dosing algorithms as Class II medical device software under the SaMD framework. Manufacturers must submit a 510(k) premarket notification demonstrating substantial equivalence to existing cleared devices. The submission requires clinical validation data from at least two independent sites with a minimum of 200 patients each, evidence of cybersecurity controls meeting FDA premarket guidance, and documentation of clinical decision support features that prevent automation bias.

The FDA, Health Canada, and the UK’s Medicines and Healthcare Products Regulatory Agency (MHRA) have jointly identified 10 guiding principles for Good ML Practice, emphasising the importance of clinical validation, transparency, and continuous monitoring [38]. Warfarin dosing algorithms must define acceptable performance thresholds, typically maintaining MAE within ±0.5 mg/day and time in therapeutic range above 65%. Modifications requiring new 510(k) submission include changes to intended use population, fundamental algorithm architecture, or clinical decision thresholds.

Healthcare systems implementing ML-guided warfarin dosing must ensure their chosen algorithm has appropriate regulatory clearance and maintain compliance with post-market surveillance requirements. The dynamic nature of ML algorithms presents unique regulatory challenges, as models may evolve with additional data, potentially requiring renewed validation or regulatory review.

#### 4.6.2. Economic Considerations

The economic implications of ML-guided warfarin dosing warrant careful consideration. Whilst our systematic review demonstrates consistent clinical effectiveness of ML-guided warfarin dosing, no included studies reported implementation costs or conducted formal economic evaluations. Future research should include comprehensive cost-effectiveness analyses using real-world implementation data. The prevention of major bleeding events, which occur in 0.4% to 7.2% of warfarin patients annually [11], could result in significant cost savings. Warfarin-related problems account for approximately 15% of all drug-related emergency department visits [12], suggesting substantial healthcare resource utilisation. Similarly, the prevention of thromboembolic events, with their associated long-term disability and care requirements, could provide additional economic justification for implementation.

The economic case for ML-guided warfarin dosing rests primarily on preventing adverse events. First-year incremental costs for warfarin-related adverse events in Medicare patients include USD 32,900 for ischaemic stroke, USD 23,414 for major bleeding, and USD 47,640 for intracranial haemorrhage [39].

Based on the demonstrated improvements from our systematic review, preventing adverse events through ML-guided dosing could generate significant savings. Zeng et al. [20] showed the safety responder ratio improved from 83.1% to 99.5%, representing a 16.4% absolute risk reduction. With major bleeding events costing USD 23,414 each, preventing 16 events per 1000 patients annually would save approximately USD 374,624. Similarly, reducing time to therapeutic INR from 4.73 to 3.77 days could decrease initial hospitalisation costs and reduce exposure to subtherapeutic or supratherapeutic INR levels.

Published estimates for healthcare ML implementation range from USD 40,000 for basic AI functionality to over USD 100,000 for comprehensive solutions [40]. For warfarin management specifically, implementation costs would likely fall in the lower range given the focused application and existing EHR infrastructure, with cloud-based solutions potentially reducing initial investment further.

However, the initial investment required for implementation should not be underestimated. Costs include not only the technical infrastructure but also staff training, workflow redesign, and ongoing quality assurance. The development of cloud-based solutions and mobile applications, as demonstrated by Amruthlal et al. [32], may reduce the barrier to entry for resource-limited settings. A formal cost-effectiveness analysis comparing different implementation strategies would provide valuable guidance for healthcare systems considering adoption of these technologies.

#### 4.6.3. Adaptation for Resource-Limited Settings

Mobile health technologies offer potential solutions for resource-constrained environments where warfarin remains the only affordable anticoagulant. Amruthlal et al. [32] demonstrated feasibility with their mobile application, achieving R^2^ of 0.955 in Indian patients, though this required population-specific development. Cloud-based algorithms could provide sophisticated dosing support without requiring local computational infrastructure, as shown by Dai et al. [30] during COVID-19. However, successful adaptation requires more than technical solutions. Algorithms must account for irregular INR monitoring common in resource-limited settings, where patients may travel hours for testing. They must also handle incomplete data, as genetic testing and comprehensive laboratory panels are often unavailable. Most critically, algorithms need training on local dietary patterns affecting vitamin K intake, local drug availability, and endemic conditions affecting warfarin metabolism. Until these adaptations occur, impressive results from well-resourced Chinese hospitals cannot be safely applied to rural clinics in sub-Saharan Africa or South America, where the need for improved warfarin management is arguably greatest.

### 4.7. Model Interpretability

A “black box” model is an ML programme where you cannot easily understand how it makes its decisions. Whilst deep neural networks and complex combined methods often achieve high accuracy, they provide predictions without explaining their reasoning. This lack of transparency creates significant challenges in clinical practice, where understanding medical decisions is essential for patient safety and gaining doctors’ trust [41].

Doctors need to understand why a programme recommends a specific dose to check if it makes sense, identify potential errors, and learn from the decision process. When an ML programme suggests an unusual warfarin dose, healthcare providers need to know whether it is based on genetic factors, kidney function, drug interactions, or other clinical variables. Without this transparency, even highly accurate programmes may not be trusted or used, and opportunities for clinical learning are lost [42]. Shapley additive explanations (SHAP), developed by Lundberg and Lee [43], calculate how much each patient characteristic contributes to the final dose prediction. Local interpretable model-agnostic explanations (LIME), created by Ribeiro et al. [44], explain individual predictions by creating a simpler model that approximates the complex model for that specific patient. These tools help transform unclear predictions into transparent recommendations, showing clinicians exactly which factors led to each warfarin dose suggestion. This increases trust and enables better clinical decision making.

Three studies in our review (21.4%) incorporated interpretability features, though implementation varied significantly. Guo et al. employed SHAP analysis, revealing that previous INR values contributed 42% to dose predictions, followed by age (18%), concurrent medications (15%), and body weight (12%). This transparency enabled clinicians to understand when the algorithm heavily weighted recent INR instability, prompting closer monitoring.

Choi et al. used LIME to explain individual predictions, discovering that their RF model inappropriately weighted dietary vitamin K intake in 23% of cases where dietary information was outdated. This finding led to implementation of data freshness requirements, accepting only dietary assessments within 30 days. Dryden et al. implemented a simpler feature importance display showing the top five factors for each prediction, though without quantitative contribution scores.

The absence of interpretability features in 11 studies (78.6%) represents a critical implementation barrier. Clinicians cannot verify algorithmic reasoning without understanding which factors drive predictions. For warfarin dosing, interpretability must identify when algorithms make predictions based on outdated information, missing genetic data, or drug interactions not present in training data. Future implementations should mandate real-time SHAP values displaying percentage contribution of each factor, with alerts when any single factor contributes more than 40% to the prediction.

### 4.8. Potential Harms and Unintended Consequences

Whilst ML algorithms demonstrate statistical improvements in controlled research settings, their implementation in clinical practice presents multiple scenarios with significant potential for patient harm. The transition from research to practice introduces complexities and risks that are often underestimated in the current literature.

Over-reliance on algorithmic recommendations represents one of the most insidious risks. Healthcare providers, particularly those with less experience or in high-pressure environments, may defer to algorithm suggestions even when clinical intuition suggests otherwise. Consider a patient presenting with subtle signs of occult bleeding such as unexplained fatigue, mild pallor, or vague abdominal discomfort that are not yet reflected in laboratory data. The algorithm, seeing only a low INR value, might recommend a dose increase to achieve therapeutic range. However, an experienced clinician would recognise these subtle clinical signs and either maintain the current dose or potentially reduce it whilst investigating for sources of bleeding. Following the algorithmic recommendation in this scenario could precipitate a major bleeding event that clinical judgement would have prevented.

Several specific scenarios exist where traditional dosing methods may prove superior to algorithmic approaches. Patients with rare genetic variants in CYP2C9 or VKORC1 genes that were not represented in training datasets present particular challenges. These patients may have dramatically different warfarin metabolism patterns that the algorithm cannot predict accurately. Traditional careful dose titration with close monitoring, though slower, may be significantly safer than algorithmic predictions based on different genetic profiles. The algorithm’s confidence in its prediction might mask the uncertainty inherent in extrapolating to these rare variants.

Complex drug interactions present another domain where clinical expertise may surpass algorithmic predictions. When patients begin taking new medications or supplements that were not present in the training dataset, experienced clinicians can anticipate potential interactions based on their understanding of pharmacological principles and metabolic pathways. Algorithms, however, cannot extrapolate beyond their training data and may fail to recognise novel drug interactions. This limitation becomes particularly dangerous with the continuous introduction of new medications and the increasing use of herbal supplements that may not be documented in EHR.

### 4.9. Future Research Priorities

Several critical research gaps must be addressed to advance the field. The most pressing need is for large-scale, multicentre randomised controlled trials comparing ML-guided dosing to standard care. These trials should be powered to detect differences in clinical safety outcomes (bleeding, thromboembolism, mortality), not merely surrogate markers such as time in therapeutic range. Without this fundamental evidence, all other research activities—however sophisticated—remain academic exercises rather than clinically actionable advances. The ongoing digitalisation of healthcare systems and increasing availability of EHR make such trials increasingly feasible.

An international warfarin algorithm benchmarking consortium should maintain a reference dataset of patients with standardised performance metrics. All studies should report MAE in mg/day, percentage within 20% of target dose, and time in therapeutic range at specified intervals. Calibration assessment through Brier scores and calibration plots is mandatory. Publications must comply with Transparent Reporting of a Multivariable Prediction Model for Individual Prognosis or Diagnosis-AI (TRIPOD-AI) guidelines [45] and register algorithms before validation studies commence.

The development of algorithms that can explicitly handle missing data and uncertain information represents another important research priority. Clinical practice is characterised by incomplete information, and algorithms that require complete data may have limited real-world applicability. The work by Wani et al. [28] using generative adversarial networks for imputation represents a promising direction, but more research is needed to validate these approaches in prospective clinical settings.

Future research must prioritise a large-scale adaptive platform trial to definitively establish the safety and efficacy of ML-guided warfarin dosing. Based on our analysis showing major bleeding rates ranging from 0.4% to 7.2% annually [11], detecting a clinically meaningful 25% relative reduction in adverse events requires enrolment of 7000 patients, with 3500 patients randomised to each arm. This calculation assumes a 2% annual major bleeding rate and accounts for an expected 20% attrition rate over the study period. For composite endpoints that include both bleeding and thrombotic events, a sample size of 5000 patients would provide adequate statistical power given the higher combined event rates.

According to ethnic differences, each ethnic population requires prospective validation in at least 1000 patients with 500 additional patients for external validation. Special populations need dedicated studies including 2000 elderly patients and 1000 patients with renal impairment. Long-term safety surveillance requires an international registry with mandatory adverse event reporting within 48 h and five-year minimum follow-up. All algorithms must demonstrate robustness to missing data and include explainability features using SHAP or LIME methods to support clinical decision making.

A global warfarin dosing alliance should coordinate four critical initiatives. African institutions should lead development of population-specific algorithms for African patients. The European Medicines Agency and FDA should create federated learning infrastructure enabling privacy-preserving algorithm improvement. World Health Organisation (WHO) Collaborating Centres should develop implementation toolkits for resource-limited settings. The Pharmacogenomics Knowledgebase (PharmGKB) consortium should integrate pharmacogenomic data into dosing algorithms.

The integration of ML with emerging technologies, particularly continuous INR monitoring devices and pharmacogenomic testing, offers exciting possibilities for personalised anticoagulation management. As continuous monitoring devices become available, algorithms will need to be adapted to handle streaming data rather than discrete measurements. Similarly, the declining cost of genetic testing may make routine pharmacogenomic profiling feasible, providing additional inputs for algorithmic dosing.

## 5. Conclusions

This systematic review identifies promising preliminary evidence that ML algorithms may improve warfarin dosing accuracy in specific research settings. However, the current evidence base—characterised by a fundamental absence of adequate safety outcome data, with only 21.4% of studies reporting safety data and none powered to detect clinically meaningful differences, retrospective designs (78.6%), limited external validation (50%), and geographic concentration (43% from China)—cannot support recommendations for clinical implementation.

Critical research priorities must be addressed before clinical adoption can be considered: (1) multicentre prospective trials powered for safety outcomes in diverse populations; (2) validation studies specifically assessing performance across ethnic groups, particularly in currently unrepresented African and South American populations; (3) development of interpretable models that clinicians can understand and override when necessary; (4) real-world implementation studies with adequate sample sizes to detect rare adverse events; (5) regulatory frameworks for continuous learning algorithms; and (6) economic evaluations including implementation costs and liability considerations.

The path forward requires acknowledging that impressive performance metrics in retrospective analyses do not equate to clinical readiness. Healthcare systems considering implementation should proceed cautiously, maintaining human oversight, conducting thorough local validation, and implementing robust safety monitoring.

## Figures and Tables

**Table 1:** Characteristics of included studies.

Study	Country/Region	Study Design	Sample Size (*n*)	Model Type
Zeng et al. [20]	China	Retrospective cohort study	10,408	Development
Ji et al. [21]	United States	Retrospective cohort study	12,497	Development with external validation
Petch et al. [22]	Multinational (52 countries)	Retrospective cohort study	28,232	Development with external validation
Guo et al. [23]	China	Retrospective cohort study	413	Development
Ganji et al. [24]	Iran	Cross-sectional study	490	Development
Wang et al. [25]	China	Retrospective cohort study	641	Development
Choi et al. [26]	South Korea, United States	Retrospective cohort study	3168 (+891 validation)	Development with external validation
Kuang et al. [27]	China	Prospective cohort study	624 (+158 validation)	Development with external validation
Wani et al. [28]	United States	Retrospective cohort study	61,532	Development
Dryden et al. [29]	Canada	Retrospective cohort study	1031	Development with internal validation
Dai et al. [30]	China	Retrospective cohort study	241	Development
Bontempi et al. [31]	Italy	Retrospective cohort study	1796	Development with external validation
Amruthlal et al. [32]	South India	Retrospective cohort study	1092	Development with external validation
Xue et al. [33]	China	Prospective cohort study	246	Development with external validation

**Table 2:** ML vs. clinical/control performance in warfarin dosing.

Study	Outcome Metric	ML	Clinical/Control	Improvement	*p*-Value	95% CI/Statistical Measure
CLINICAL OUTCOMES						
Zeng et al. [20]	Excellent responder ratio	RL: 80.8%	Clinicians: 41.6%	+94.2%	-	RR: 0.51 (95% CI: 0.48–0.55)
	Safety responder ratio	RL: 99.5%	Clinicians: 83.1%	+19.7%	-	RR: 0.83 (95% CI: 0.81–0.86)
	Target responder ratio	RL: 81.1%	Clinicians: 49.7%	+63.2%	-	RR: 0.61 (95% CI: 0.58–0.65)
	Time to target INR (days)	RL: 3.77	Clinicians: 4.73	−20.3%	-	AD: 0.96 (95% CI: 0.84–1.08)
	Time in target range (days)	RL: 4.88	Clinicians: 2.57	+89.9%	-	AD: 2.31 (95% CI: 2.06–2.56)
Petch et al. [22]	TTR improvement per 10% adherence	RL: 6.78%	Benchmark: 6.10%	+11.1%	*p* < 0.001	95% CI: 6.29–7.28
			Benchmark		*p* < 0.001	95% CI: 5.67–6.54
	Composite outcome (HR)	RL: 11% decrease	Benchmark: 10% decrease	+10.0%	*p* = 0.015	HR: 0.89 (95% CI: 0.81–1.00)
					*p* = 0.018	HR: 0.90 (95% CI: 0.83–0.98)
IMPLEMENTATION OUTCOMES						
Dai et al. [30]	Good anticoagulation quality	IAC (ML): 69.8%	HAC: 73.1%	−4.5%	*p* = 0.576	TTR: IAC 80.6 ± 21.1%, HAC 79.9 ± 20.0%
	Major bleeding	IAC: 1.0%	HAC: 0.69%	+44.9%	*p* = 1.000	-
	CRNMB	IAC: 40.6%	HAC: 39.3%	+3.3%	*p* = 0.838	-
	Thromboembolic events	IAC: 1.0%	HAC: 1.4%	−28.6%	*p* = 1.000	-
Dryden et al. [29]	Therapeutic INR at discharge	Post-ML: 61.1% (11/18)	Pre-ML: 47.5% (305/641)	+28.6%	*p* = 0.37	Non-significant

Abbreviations: AD, absolute difference; CI, confidence interval; CRNMB, clinically relevant non-major bleeding; HAC, hospital-based anticoagulation clinic; HR, hazard ratio; IAC, internet-based anticoagulation clinic; INR, international normalized ratio; ML, machine learning; RL, reinforcement learning; RR, relative risk; TTR, time in therapeutic range.

**Table 3:** Geographic distribution of studies.

Region	Number of Studies	Total Sample Size	Percentage of Total
China (all regions)	6	12,573	10.3%
United States	2	74,029	60.5%
South Korea/United States	1	4059	3.3%
India	1	1092	0.9%
Italy	1	1796	1.5%
Canada	1	1031	0.8%
Iran	1	490	0.4%
Multinational	1	28,232	23.1%
Total	14	122,411	100%

**Table 4:** Comparison of ML algorithms with clinical practice—Zeng et al. [20].

Algorithm	Excellent Responder Ratio	Improvement vs. Clinical Practice	Safety Responder Ratio	Target Responder Ratio
Clinical Practice	41.6%	Baseline	83.1%	49.7%
RL	80.8%	+94.2%	99.5%	81.1%
LSTM	77.1%	+85.3%	98.7%	78.3%
ANN	75.0%	+80.3%	98.5%	76.0%
Rule-Based (RB)	67.2%	+61.5%	96.8%	68.7%
EEM	67.6%	+62.5%	97.9%	69.3%
SVM	66.4%	+59.6%	98.0%	67.9%

Abbreviations: RL, reinforcement learning; LSTM, long short-term memory; ANN, artificial neural network; SVM, support vector machine; EEM, evolutionary ensemble model.

**Table 5:** PROBAST risk of bias assessment.

Study	Participants Risk	Predictors Risk	Outcome Risk	Analysis Risk	Overall Risk of Bias	Overall Applicability
Amruthlal et al. [32]	LOW	LOW	LOW	UNCLEAR-probably high	UNCLEAR-probably high	LOW
Bontempi et al. [31]	LOW	LOW	LOW	LOW	LOW	LOW
Dai et al. [30]	LOW	LOW	LOW	HIGH	HIGH	LOW
Dryden et al. [29]	LOW	LOW	LOW	UNCLEAR-probably high	UNCLEAR-probably high	LOW
Guo et al. [23]	UNCLEAR-probably low	LOW	LOW	HIGH	HIGH	UNCLEAR-probably high
Choi et al. [26]	LOW	LOW	LOW	UNCLEAR-probably high	UNCLEAR-probably high	UNCLEAR-probably low
Ji et al. [21]	LOW	LOW	LOW	HIGH	HIGH	UNCLEAR-probably high
Kuang et al. [27]	LOW	LOW	UNCLEAR-probably low	UNCLEAR-probably high	UNCLEAR-probably high	LOW
Petch et al. [22]	LOW	LOW	LOW	LOW	LOW	LOW
Wang et al. [25]	LOW	LOW	LOW	LOW	LOW	LOW
Xue et al. [33]	LOW	UNCLEAR-probably low	LOW	UNCLEAR-probably low	UNCLEAR-probably low	LOW
Zeng et al. [20]	LOW	LOW	LOW	UNCLEAR-probably high	UNCLEAR-probably high	LOW
Wani et al. [25]	HIGH	LOW	LOW	HIGH	HIGH	UNCLEAR-probably high
Ganji et al. [24]	LOW	LOW	LOW	UNCLEAR-probably high	UNCLEAR-probably high	UNCLEAR-probably high

**Table 6:** Comparative analysis of ML algorithms for warfarin dosing.

Algorithm Type	Advantages	Disadvantages	Best Use Case	Study Performance
Reinforcement Learning *n* = 10,408−28,232	•Learns from outcomes •Adapts to individual responses • Handles sequential decisions • Self-improving	• Requires extensive data • Black box predictions • Complex implementation • Difficult regulatory path	Dynamic dose adjustment in frequently monitored patients	BCQ: 98.55% accuracy RL: 80.6% vs 41.6% responder ratio
Deep Learning (LSTM/NN) *n* = 624−19,719	• Captures complex patterns • Excellent for time-series • Automatic feature learning	• Large data requirement • Not interpretable • Prone to overfitting • High computational cost	INR prediction with rich temporal data	LSTM: 70% accuracy DNN: 84% within ±0.3 INR
Tree-Based (RF/XGBoost) *n* = 246−3168	• Feature importance available • Handles missing data • Robust to outliers • Mixed data types OK	• Can overfit small samples • Limited extrapolation • Memory intensive • Requires tuning	Stable dose prediction with clinical/genetic data	XGBoost: MAE 0.9 mg RF: 80.56% within 20%
SVM *n* = 413−1092	• Good with limited samples • Strong generalization • Robust to overfitting	• Kernel selection critical • Black box (non-linear) • No probability output • Slow for large data	Homogeneous populations with moderate samples	R^2^ = 0.98 MAE: 0.14 mg/day
Ensemble Methods *n =* 641−1031	• Combines model strengths • Reduces individual bias • Often best accuracy	• Increased complexity • Difficult to interpret • Longer training time • Complex deployment	Production systems requiring maximum accuracy	Stacking: 73.44% accuracy R^2^ = 0.87
Traditional ML (Linear) *n* = 156−4798	• Fully interpretable • Minimal data needed • Fast and stable • Clear regulatory path	• Cannot capture non-linearity • Limited interactions • Lower accuracy ceiling • Sensitive to outliers	Resource-limited settings, baseline models	R^2^ = 0.454 MAE: 8.1−8.3 mg/week

Machine Learning Algorithms for Anticoagulation Management. Abbreviations: BCQ, batch-constrained Q-learning; DNN, deep neural network; INR, international normalized ratio; LSTM, long short-term memory; MAE, mean absolute error; ML, machine learning; NN, neural network; R^2^, coefficient of determination; RF, random forest; RL, reinforcement learning; XGBoost, extreme gradient boosting.

## Data Availability

No new data were created or analyzed in this study. Data sharing is not applicable to this article.

## Supplementary Materials

The following supporting information can be downloaded at: https://www.mdpi.com/article/10.3390/ph18101544/s1, Table S1: Prisma check list; Table S2: Machine Learning vs. Clinical/Control Performance in Warfarin Dosing. Table S3: Best Results by Algorithm Type.

## Abbreviations

Abbreviation	Full Term
AD	Absolute Difference
AI	Artificial Intelligence
ANCOVA	Analysis of Covariance
ANN	Artificial Neural Network
ARR	Absolute Risk Reduction
AUC	Area Under Curve
BCQ	Batch-Constrained Q-Learning
CI	Confidence Interval
CRNMB	Clinically Relevant Non-Major Bleeding
CYP2C9	Cytochrome P450 2C9
DOACs	Direct Oral Anticoagulants
E2GAN	Enhanced Generative Adversarial Network
EEM	Evolutionary Ensemble Model
EHR	Electronic Health Records
FDA	Food and Drug Administration
GAIN	Generative Adversarial Imputation Nets
GRU-D	Gated Recurrent Unit with Decay
HAC	Hospital-based Anticoagulation Clinic
HR	Hazard Ratio
IAC	Internet-based Anticoagulation Clinic
INR	International Normalised Ratio
IT	Information Technology
IWPC	International Warfarin Pharmacogenetics Consortium
LIME	Local Interpretable Model-agnostic Explanations
LSTM	Long Short-Term Memory
MAE	Mean Absolute Error
MAPB	Maximum A Posteriori Bayesian
MHRA	Medicines and Healthcare products Regulatory Agency
MICE	Multivariate Imputation by Chained Equations
ML	Machine Learning
MLR	Multiple Linear Regression
NNT	Number Needed to Treat
PharmGKB	Pharmacogenomics Knowledgebase
PRISMA	Preferred Reporting Items for Systematic Reviews and Meta-Analyses
PROBAST	Prediction model Risk of Bias Assessment Tool
R^2^	Coefficient of Determination
RB	Rule-Based
RF	Random Forest
RL	Reinforcement Learning
RMSE	Root Mean Square Error
RR	Relative Risk
SAM	Semi-empirical Anticoagulation Model
SaMD	Software as Medical Device
SHAP	Shapley Additive Explanations
SVM	Support Vector Machine
SWiM	Synthesis Without Meta-analysis
TRIPOD-AI	Transparent Reporting of a Multivariable Prediction Model for Individual Prognosis or Diagnosis-AI
TTR	Time in Therapeutic Range
VKORC1	Vitamin K Epoxide Reductase Complex Subunit 1
WHO	World Health Organization
XGBoost	Extreme Gradient Boosting
