# Supplementary material for: Machine Learning for Warfarin Therapy: A Systematic Review
Source: Pharmaceuticals (Basel). 2025 Oct 14;18(10):1544. doi: 10.3390/ph18101544 (PMC12567301; doi:10.3390/ph18101544)
Supplement: Supplementary file 1 [file pharmaceuticals-18-01544-s001.zip › pharmaceuticals-3860075-supplementary.pdf]

Table S1. Prisma check list.

Section	Item	Checklist Item	Reported	Details from Article
TITLE	1	Identify the report as a systematic review	✓ Yes	Title: "A Systematic Review"
ABSTRACT	2	Provide structured summary including objectives, methods, results, conclusions	✓ Yes	Structured abstract present with Background, Methods, Results, Conclusions
INTRODUCTION	3	Rationale: Describe rationale in context of existing knowledge	✓ Yes	Section 1: "warfarin remains essential for mechanical valves, renal impairment, and resource-limited settings"
	4	Objectives: Provide explicit statement of objectives	✓ Yes	"This systematic review evaluates the literature on machine learning approaches for warfarin dose prediction (2022-2025)"
METHODS	5	Eligibility criteria: Specify inclusion/exclusion criteria	✓ Yes	Section 2.2: Studies using ML for warfarin dosing with quantifiable metrics, adult populations, 2022-2025
	6	Information sources: Specify all databases, registers, other sources	✓ Yes	"PubMed and Semantic Scholar databases" through August 2025
	7	Search strategy: Present full search strategies	✓ Yes	Terms included: "warfarin," "machine learning," "artificial intelligence," "dose prediction," "anticoagulation"
	8	Selection process: State process for selecting studies	✓ Yes	"Two-stage screening" with 67 records identified
	9	Data collection process: Describe data extraction methods	✓ Yes	"Two independent reviewers (P.F., T.P.) systematically extracted data"
	10a	Data items: List all outcomes sought	✓ Yes	MAE, RMSE, prediction accuracy, R^2, time in therapeutic range
	10b	Data items: List all other variables	✓ Yes	Country, study design, sample size, model type, validation approach
	11	Study risk of bias assessment	✓ Yes	"PROBAST framework" used
	12	Effect measures: Specify effect measures	✓ Yes	MAE (mg/day), R^2, prediction accuracy percentages, responder ratios
	13a	Synthesis methods: Describe eligibility for synthesis	✓ Yes	"Quantitative meta-analysis not feasible due to substantial heterogeneity"
	13b	Synthesis methods: Describe preparation methods	✓ Yes	"Narrative synthesis following SWiM reporting guidelines"
	13c	Synthesis methods: Describe tabulation/visualization	✓ Yes	Tables 1-6 present study characteristics and performance metrics
	13d	Synthesis methods: Describe synthesis methods	✓ Yes	Narrative synthesis by algorithm type, population, quality
	13e	Synthesis methods: Describe effect modifiers	✓ Yes	Geographic region, algorithm type, study quality analyzed
	13f	Synthesis methods: Describe sensitivity analyses	✗ No	Not reported
	14	Reporting bias assessment	✓ Partial	"Funnel plot analysis not possible...patterns suggest publication bias"
	15	Certainty assessment	✗ No	No GRADE or similar assessment reported
RESULTS	16a	Study selection: Give numbers at each stage	✓ Yes	"67 records...14 studies met all inclusion criteria"
	16b	Study selection: Cite excluded studies	✓ Yes	"5 studies excluded: 1 language barriers, 4 methodological limitations"
	17	Study characteristics	✓ Yes	Table 1: 14 studies, 122,411 patients, 9 countries
	18	Risk of bias in studies	✓ Yes	Table 5: 21.4% low risk, 28.6% high risk, detailed PROBAST assessment
	19	Results of individual studies	✓ Yes	Tables 2-4: Detailed performance metrics for each study
	20a	Results of syntheses	✓ Yes	MAE: 0.11-1.8 mg/day, R^2: 0.56-0.98, Accuracy: 53.9-98.55%

	20b	Results of syntheses: Statistical analyses	N/A	Meta-analysis not performed due to heterogeneity
	20c	Results of syntheses: Sensitivity analyses	✗ No	Not performed
	20d	Results of syntheses: Heterogeneity	✓ Yes	Heterogeneity discussed extensively by region, algorithm, quality
	21	Reporting biases	✓ Yes	"Complete absence of negative results, selective reporting of uncertainty"
	22	Certainty of evidence	✗ No	No formal certainty assessment
	23a	Risk of bias and results	✓ Yes	"Inverse relationship between study quality and reported performance"
	23b	Non-reporting biases and results	✓ Yes	Geographic concentration (43% China), missing populations discussed
	23c	Certainty and results	✗ No	Not assessed
	23d	Heterogeneity and results	✓ Yes	Section 3.6: Detailed subgroup analyses
DISCUSSION	24a	General interpretation	✓ Yes	Section 4.1: "Most critical finding...absence of robust safety data"
	24b	Limitations of evidence	✓ Yes	Section 4.2: Retrospective bias (78.6%), validation crisis, sample size issues
	24c	Limitations of review	✓ Yes	"Not prospectively registered in PROSPERO" acknowledged
	24d	Implications	✓ Yes	Section 4.9: Future research priorities detailed
OTHER	25	Registration	✓ Yes	"Not prospectively registered in PROSPERO" - transparently reported
	26	Support	✓ Yes	"Funded by VEGA, grant number 1/0700/23"
	27a	Competing interests	✓ Yes	"Authors declare no direct conflicts of interest"
	27b	Other information	✓ Yes	Supplementary materials available

Table S2. Machine Learning vs Clinical/Control Performance in Warfarin Dosing

Study	Outcome Metric	Machine Learning	Clinical/Control	Improvement	p-value	95% CI / Statistical Measure
			DOSE PREDICTION ACCURACY			
Guo et al. [23]	R^2 value	SVM: 0.98	IWPC: 0.43	+127.9%	–	SVM MAE 95% CI: 0.11–0.17 mg/day
	MAE	SVM: 0.14 mg/day	IWPC: 8.5 mg/week	−98.4%	–	95% CI: 0.11–0.17
	Ideal prediction – Low dose	SVM: 85.71%	IWPC: 33.00%	+159.7%	–	–
	Ideal prediction – Medium dose	SVM: 95.92%	IWPC: 54.60%	+75.6%	–	–
	Ideal prediction – High dose	SVM: 92.00%	IWPC: 36.80%	+150.0%	–	–
Choi et al. [26]	MAE (internal validation)	XGBoost: 0.9 mg	Physicians: 1.3 mg	−30.8%	–	–
		ANN: 0.9 mg	Physicians: 1.3 mg	−30.8%	–	–
		Random Forest: 1.0 mg	Physicians: 1.3 mg	−23.1%	–	–
	Accuracy (internal)	ML models: 50%	Physicians: 23%	+117.4%	–	–
	MAE (external validation)	RF: 1.8 mg	Physicians: 1.8 mg	0 %	–	–
		XGBoost: 1.8 mg	Physicians: 1.8 mg	0 %	–	–
		ANN: 2.0 mg	Physicians: 1.8 mg	+11.1%	–	–
Bontempi et al. [31]	Dose prediction error	SAM: 3.24 ± 25.80%	Literature: 5.73 ± 60.9%	−43.4%	Significant	Significantly more accurate
Kuang et al. [27]	Overall prediction accuracy	LSTM: 70.0%	MAPB: 53.9%	+29.9%	$p < 0.05$	–
	With temporal variables	LSTM: 70.0%	Without: 51.7%	+35.4%	$p < 0.05$	–
	With genetic factors	LSTM: 70.0%	Without: 61.5%	+13.8%	$p < 0.05$	–
	Accuracy within ±20%	LSTM: 53.93%	–	–	–	–
Ji et al. [21]	Accuracy within ±20%	BCQ (τ=0.8): 98.55%	XGBoost: 64.07%	+53.7%	–	–
		BCQ (τ=0.5): 97.65%	XGBoost: 64.07%	+52.3%	–	–
		BCQ (τ=0.3): 96.96%	XGBoost: 64.07%	+51.3%	–	–
	Accuracy within ±15%	BCQ (τ=0.8): 98.32%	XGBoost: 49.00%	+100.7%	–	–
	Accuracy within ±5%	BCQ (τ=0.8): 94.55%	XGBoost: 40.15%	+135.5%	–	–
Ganji et al. [24]	Accuracy (internal)	ensemble: MLR, SVM, RF 76.4%	MLR: 70.9%	+7.2%	–	–

Abbreviations: ANN, artificial neural network; BCQ, batch-constrained Q-learning; CI, confidence interval; IWPC, International Warfarin Pharmacogenetics Consortium; LSTM, long short-term memory; MAE, mean absolute error; MAPB, maximum a posteriori Bayesian; ML, machine learning; MLR, multiple linear regression; R^2, coefficient of determination; RF, random forest; SAM, semi-empirical anticoagulation model; SVM, support vector machine; XGBoost, extreme gradient boosting.

Table S3. Best Results by Algorithm Type.

Algorithm	Best Result	Study	Metric	Population (*n*)
RL	98.6%	Ji et al. [21]	Prediction accuracy	12,497
	80.8% vs 41.6%	Zeng et al. [20]	Excellent responder ratio	10,408
	$R^2 = 0.56$	Petch et al. [22]	R-squared	28,232
RF	MAE = 0.9 mg	Choi et al. [26]	Mean Absolute Error	3168
	MAE = 1.13 mg	Dryden et al. [29]	Mean Absolute Error	1031
	Correlation = 0.978 (training)	Xue et al. [33]	Correlation coefficient	246
XGBoost	MAE = 0.9 mg	Choi et al. [26]	Mean Absolute Error	3168
	AUC = 0.808	Dai et al. [30]	Area Under Curve	241
SVM	$R^2 = 0.955$	Amruthlal et al. [32]	R-squared	1092
	$R^2 = 0.98$	Guo et al. [23]	R-squared	413
LSTM	70.0%	Kuang et al. [27]	Prediction accuracy	624
	77.1%	Zeng et al. [20]	Excellent responder ratio	10,408
Ensemble Methods	$R^2 = 0.87$	Wang et al. [25]	R-squared	641
	MAE = 1.11 mg	Dryden et al. [29]	Mean Absolute Error	1031
Deep Learning (GAN/DAE)	20–35% RMSE reduction	Wani et al. [28]	RMSE improvement	61,532

Abbreviations: AUC, area under curve; DAE, denoising autoencoders; GAN, generative adversarial network; LSTM, long short-term memory; MAE, mean absolute error; R^2, coefficient of determination; RF, random forest; RL, reinforcement learning; RMSE, root mean square error; SVM, support vector machine; XGBoost, extreme gradient boosting.
